# Physical mapping of a large plant genome using global high-information-content-fingerprinting: the distal region of the wheat ancestor *Aegilops tauschii *chromosome 3DS

**DOI:** 10.1186/1471-2164-11-382

**Published:** 2010-06-17

**Authors:** Delphine Fleury, Ming-Cheng Luo, Jan Dvorak, Luke Ramsay, Bikram S Gill, Olin D Anderson, Frank M You, Zahra Shoaei, Karin R Deal, Peter Langridge

**Affiliations:** 1 Australian Centre for Plant Functional Genomics, University of Adelaide, PMB1, Glen Osmond SA 5064, Australia; 2 Department of Plant Sciences, University of California, Davis, CA 95616, USA; 3 Genetics Programme, Scottish Crop Research Institute, Invergowrie, Dundee DD2 5DA, Scotland, UK; 4 Department of Plant Pathology, Kansas State University, Manhattan KS 66506, USA; 5 Genomics and Gene Discovery Unit, U.S. Department of Agriculture/Agricultural Research Service Western Regional Research Center, Albany, CA 94710, USA

## Abstract

**Background:**

Physical maps employing libraries of bacterial artificial chromosome (BAC) clones are essential for comparative genomics and sequencing of large and repetitive genomes such as those of the hexaploid bread wheat. The diploid ancestor of the D-genome of hexaploid wheat (*Triticum aestivum*), *Aegilops tauschii*, is used as a resource for wheat genomics. The barley diploid genome also provides a good model for the *Triticeae *and *T. aestivum *since it is only slightly larger than the ancestor wheat D genome. Gene co-linearity between the grasses can be exploited by extrapolating from rice and *Brachypodium distachyon *to *Ae. tauschii *or barley, and then to wheat.

**Results:**

We report the use of *Ae. tauschii *for the construction of the physical map of a large distal region of chromosome arm 3DS. A physical map of 25.4 Mb was constructed by anchoring BAC clones of *Ae. tauschii *with 85 EST on the *Ae. tauschii *and barley genetic maps. The 24 contigs were aligned to the rice and *B. distachyon *genomic sequences and a high density SNP genetic map of barley. As expected, the mapped region is highly collinear to the orthologous chromosome 1 in rice, chromosome 2 in *B. distachyon *and chromosome 3H in barley. However, the chromosome scale of the comparative maps presented provides new insights into grass genome organization. The disruptions of the *Ae. tauschii*-rice and *Ae. tauschii*-*Brachypodium *syntenies were identical. We observed chromosomal rearrangements between *Ae. tauschii *and barley. The comparison of *Ae. tauschii *physical and genetic maps showed that the recombination rate across the region dropped from 2.19 cM/Mb in the distal region to 0.09 cM/Mb in the proximal region. The size of the gaps between contigs was evaluated by comparing the recombination rate along the map with the local recombination rates calculated on single contigs.

**Conclusions:**

The physical map reported here is the first physical map using fingerprinting of a complete *Triticeae *genome. This study demonstrates that global fingerprinting of the large plant genomes is a viable strategy for generating physical maps. Physical maps allow the description of the co-linearity between wheat and grass genomes and provide a powerful tool for positional cloning of new genes.

## Background

Although wheat is a major food for the world population and the most extensively grown crop, progress in genomics had been slowed due to the size and the complexity of the genome. The hexaploid genome of common wheat (*Triticum aestivum*) contains 16,000 Mb of DNA organized into three genomes, A, B and D, with 7 chromosomes each. This makes the wheat genome far larger than the sequenced rice genome at 430 Mb [1] and *Brachypodium *genome at 271 Mb [2]. *T. aestivum *evolved via two hybridization events. The first event combined the A and B genomes into tetraploid wheat while the second hybridization event, which took place only about 8,500 years ago [3] and involved tetraploid wheat and diploid *Ae. tauschii *[4], contributed the D genome. Due to the recent origin of *T. aestivum *and limited gene-flow from *Ae. tauschii *[5], the D genome shows low polymorphism, and the genetic maps of the D genome chromosomes still tend to be poor.

Physical mapping strategies employing libraries of bacterial artificial chromosome (BAC) clones can potentially generate maps of a genome without extensive polymorphism and facilitate studying the structure of the genome, comparison with other genomes and map-based cloning of genes. Physical maps are also the prerequisite step for the sequencing and assembly of large and repetitive genomes such as those of wheat. Most physical mapping in wheat has generally targeted small regions covered by a few BAC clones. Two recent studies presenting physical map of the wheat chromosome 3B [6] and the short arm of chromosome 3AS and 3DS [7] exemplify a strategy adopted by the International Wheat Genome Sequencing Consortium http://www.wheatgenome.org/ for physical mapping of the 21 wheat chromosomes. The strategy makes use of BAC libraries constructed from DNA of single chromosomes or single chromosome arms isolated by chromosome or chromosome arm flow sorting [8]. A complementary approach is to construct physical maps of wheat diploid relatives and use the maps for the construction of the physical maps of the wheat chromosomes. Of the three diploid ancestors of *T. aestivum*, this strategy is the most applicable to *Ae. tauschii *because of high homology between its chromosomes and those of the wheat D genome. To further enhance the utility of *Ae. tauschii *physical map for using it as a resource for wheat D-genome genomics, an accession from the presumptive area of the origin of *T. aestivum *[5] showing a short genetic distance to the wheat D genome was used for the construction of *Ae. tauschii *BAC libraries [9] and BAC contigs [10]. This accession has also been used recently for the construction of extensive comparative map of *Ae. tauschii *based on ESTs (expressed-sequence-tags) with the rice and sorghum genome sequences [11].

The barley diploid genome is only slightly larger than the wheat D genome and, like *Ae. tauschii*, genetic maps are well populated with molecular markers making it another good model for the *Triticeae *and *T. aestivum*. Barley and *Ae. tauschii *comparative mapping have shown that the grass genomes are highly collinear and gene order is conserved across chromosomes [11,12]. Gene co-linearity can be exploited by extrapolating the knowledge obtained on simpler model species to more complex species: from rice or sorghum to *Ae. tauschii *or barley, and then from them to wheat.

Here we report the construction of the physical map of a large portion of *Ae. tauschii *chromosome arm 3DS delineated by an X-ray induced deletion mutation called *pairing homoeologous 2a *(*ph2a*) and initially located on the distal 20 cM of the arm [13]. We describe here the assembly of *Ae. tauschii *BACs into contigs and their ordering based on the *Ae. tauschii *comparative genetic map [11]. By superimposing the physical map of the 3DS region on the *Ae. tauschii *recombination map, we estimate recombination rates along the chromosome arm and the gaps in the physical map. We also describe its synteny with *Brachypodium *and rice genomic sequences and the barley genetic map.

## Results

### Physical map of a distal region of chromosome 3DS in *Ae. tauschii*

Using the southern blot data from a previous study [13], we first identified the wheat deletion bins [14] overlapping the *ph2a *deletion. Among 53 ESTs mapped into the region deleted in *ph2a*, 8 EST mapped to 6 deletion bins of group 3 chromosomes: the telomeric bin 3AS4-0.45-1.00; the 3 bins 3BS8, 3BS9 and 3BS1 covering the chromosome arm from 0.33 to 1.00 (ratio of chromosome arm length); the bins 3DS6 and 3DS3 from position 0.24 to 1.00 [14]. The GrainGenes database http://wheat.pw.usda.gov contained a total of 591 ESTs that had been mapped to these 6 bins. These ESTs including 53 EST mapped within the *ph2a *deletion were assembled into 151 unigenes according to the DFCI wheat gene index database http://compbio.dfci.harvard.edu/tgi and used for BAC anchoring. To decrease the chance of hybridizing repeat elements, probes used for the BAC library screening contained only exon sequence. PCR products for 110 unigenes of wheat were verified by direct sequencing and used as markers. Eighty probes gave clean results by hybridization onto restricted DNA of nullisomic-tetrasomic wheat lines and were then used for hybridization to the *Ae. tauschii *BAC high density screening membranes.

Using 68 such probes identified 278 positive BAC clones. These clones had been previously assembled based on fingerprints into contigs containing 3,289 BACs http://wheatdb.ucdavis.edu/. We screened the 3,289 clones with 42 additional PCR markers which could not be used for hybridization because of the poor probe quality. After validation of marker presence on clones by PCR, 1,802 BAC clones were re-assembled into 24 contigs with a size that ranged from 188 to 3,935 kb (Table 1). The physical map covered 25.4 Mb which is about one third of the target region initially estimated to be about 80 Mb by comparison with rice genomic sequence [13]. The physical map included a total of 90 loci, composed of 85 unigenes and 5 genomic based markers (See Additional File 1). A contig contained an average of 3.7 markers (ranging from 1 to 17 markers). The genes were organized in 54 gene islands defined by the presence of at least 1 gene on one BAC. Among those, 19 different gene islands were identified with at least 2 genes and up to 5 genes on a single clone.

**Table 1:** Characteristics of the Ae. tauschii physical map of the distal region of chromosome 3DS

	Total	Average/contig	Minimum	Maximum
Contigs	24			
Clones	1802	76	8	322
Loci	90	3.7	1	17
CB*	13,355	566.4	99	2,071
kb	25,374	1,057	188	3,935

* consensus band

**Figure 1:**
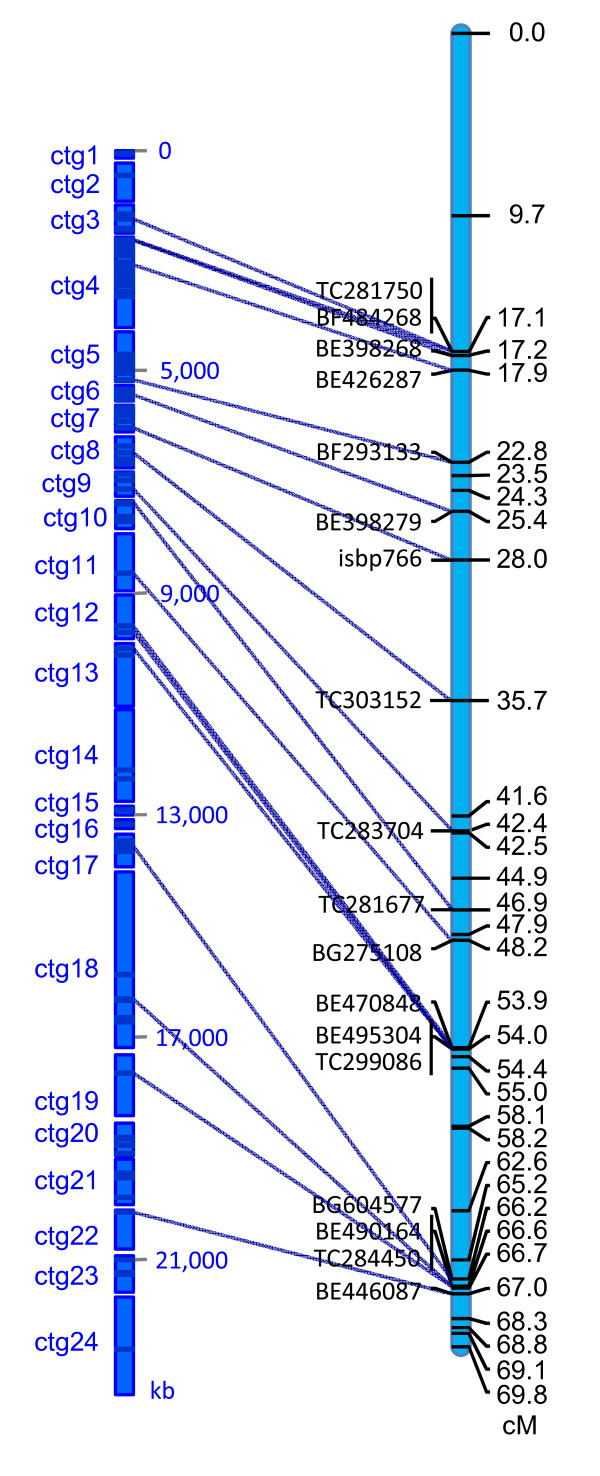
***Ae. tauschii *physical and genetic maps of chromosome 3DS**. Alignment of *Ae. tauschii *physical map onto *Ae. tauschii *genetic map of chromosome 3DS. The *Ae. tauschii *AL8/78 × AS75 F_2 _genetic map used EST based markers [11].

Due to the low polymorphism of the *T. aestivum *D genome, the wheat genetic maps are poorly populated with molecular markers and not informative enough for anchoring BAC contigs on a map. Therefore, whenever possible we have anchored the BAC contigs onto the *Ae. tauschii *genetic map built from AL8/78 × AS75 F_2 _mapping population [11]. The fact that one of the parents (AL8/78) was also used for the construction of the BAC libraries greatly assisted the anchoring of contigs on the genetic map. A contig was anchored only when a clone in the contig and a marker on the map contributed by AL8/78 had exactly the same phenotype. Eleven EST markers and one ISBP marker were used to align 14 contigs onto the *Ae. tauschii *genetic map (Figure 1 and Additional File 2). The remaining contigs were ordered as follows. Contigs (ctg) 1, 2, 21, 23 and 24 were ordered according to the 3B physical map [6]. Ctg 13, 14 and 20 were anchored and positioned in the order of the barley genetic map. Ctg 15 and 16 could not be anchored on a *Triticeae *map and were ordered according to the order of orthologous genes in the rice genome.

### Recombination along the distal region of chromosome 3DS

Meiotic recombination activity was evaluated by calculating the recombination rate or coefficient of exchange (CE) in cM per Mb. Although the actual length of a contig can not be known without BAC sequencing, the physical size of a contig was evaluated based on the number of common bands (CB units) between BAC fingerprints. The average size of 1 CB was 1.9 kb for this *Ae. tauschii *library http://wheatdb.ucdavis.edu. The average CE along the map was 2.35 cM/Mb between the most distant markers TC281750 of ctg3 and BE446087 of ctg22 (49.9 cM and 11,187 CB) (Table 2). Local CE was also measured for two pairs of markers physically linked on the ctg4 and ctg12: respectively 2.19 and 1.42 cM/Mb. In the proximal region, we calculated the physical distance between the markers BG604577 and BE446087 by adding the size of ctg17, 18, 19, 20, 21 and 22 neglecting the gaps between them. With a genetic distance of 0.78 cM, the CE was 0.09 cM/Mb. The recombination rate decreased in *Ae tauschii *from the distal region of ctg4, through the middle region of ctg12 to the proximal ctg17-22.

**Table 2:** Coefficient of exchange (CE) along the distal region of chromosome 3DS in *Ae. tauschii*

	Delineating EST		Distance between the EST		
		
Contig	left	right	cM	kb	CE (cM/Mb)
Ctg4	BF484268	BE426287	0.79	361	2.19
Ctg12	BE495304	BE591013	0.07	49.4	1.42
Ctg17-22	BG604577	BE446087	0.78	8,214	0.09
Ctg3-22	TC281750	BE446087	49.9	21,255	2.35

### Synteny with *B. distachyon*, rice and barley

**Figure 2:**
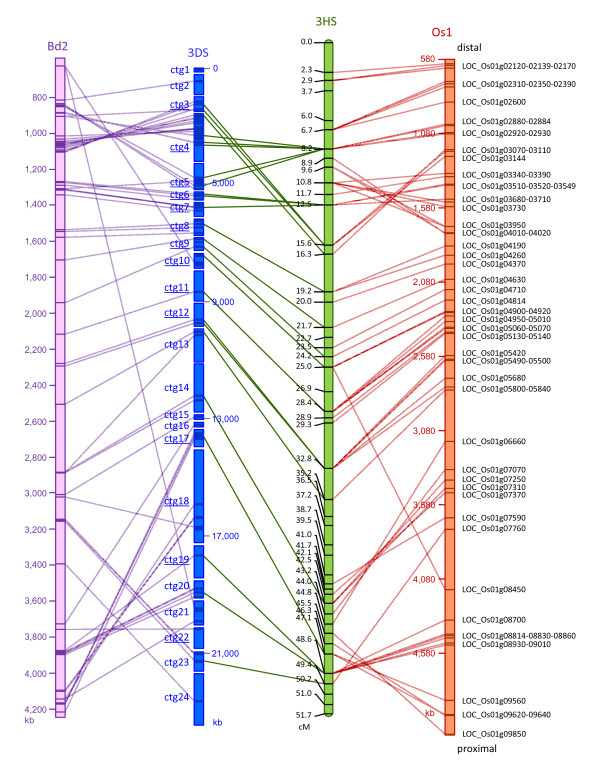
**Comparative maps of *Ae. tauschii *3DS with rice, *Brachypodium *and barley genome**. Physical map of the distal region of *Ae. tauschii *3DS compared to the orthologous genomic sequences of *B. distachyon *Bd2 (8× release) and rice Os2, and to the genetic map of barley 3HS. The underlined ctg names indicate the contigs which were aligned onto the *Ae. tauschii *AL8/78 × AS75 F_2 _genetic map [11]. The names to the right are rice genes as listed in MSU Rice Genome Annotation (Osa1) Release 6.0. The barley Steptoe x Morex genetic map used gene-based SNP [15,16].

By comparing the *Ae. tauschii *physical map of 3DS to the *B. distachyon *8× genome sequence, 53 wheat unigenes were orthologous to *B. distachyon *chromosome Bd2. A distal inversion occurred in the region between 834 and 1,105 kb of Bd2 matching the ctgs 3, 4 and 5 of *Ae. tauschii *(Figure 2). Separated by a large conserved block of about 2,035 kb in *Brachypodium*, a second large inversion has occurred in the proximal region between the ctgs 17 to 24 of *Ae. tauschii *and Bd2-3,140 and Bd2-4,211 kb. Compared to the rice genomic sequence, the *Ae. tauschii *map is highly collinear to chromosome 1 with 70 wheat unigenes of the *Ph2 *physical map orthologous to 59 rice genes of Os1 (See Additional File 1). They showed the same chromosomal rearrangements between 3DS and Os1 that have been previously described by using the same *Ae. tauschii *genetic map [11]. The synteny profile of *Ae. tauschii*-*B. distachyon *is almost identical to the one of *Ae. tauschii*-rice, showing the same inverted and conserved blocks (See Additional File 2).

A comparison was made with the genetic map of barley 3HS derived from the Steptoe x Morex doubled haploid population populated with gene-based SNP [15] and Transcript Derived Markers [16] loci. A total of 24 unigenes homologous between wheat and barley were used to integrate the *Ae. tauschii *physical map and the barley genetic map. Sixteen contigs were therefore anchored onto 15 barley genetic map positions (Figure 2). Synteny was conserved on a large block between the two species, from ctg 8 to 24. A region of 8.1 cM in length in barley has been rearranged in *Ae. tauschii *between ctgs 3, 4 and 5. This observation is supported by the anchoring of 3 markers of ctg4, which was oriented and positioned onto the *Ae. tauschii *genetic map. This rearrangement overlapped the distal rice-*Ae. tauschii *inversion previously described. It could either be due to an inversion between *Ae. tauschii *and barley leading to the translocation of ctg6 and 7 in a more distal region in barley, or to the proximal translocation of ctg 3-distal part of ctg4 in barley.

**Figure 3:**
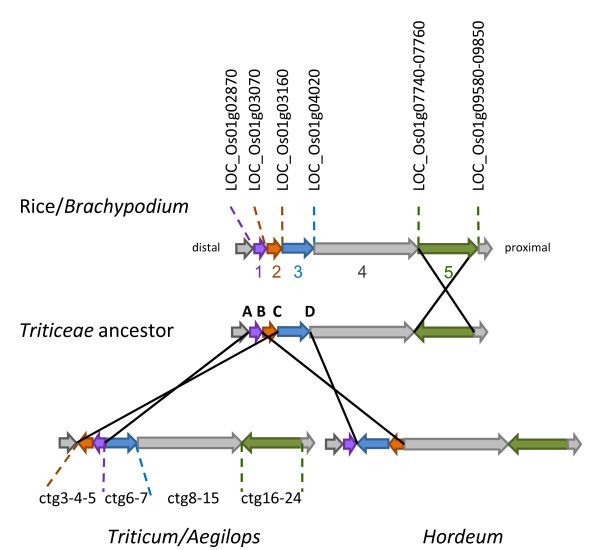
**Model of chromosomal rearrangements of the *Ph2 *locus in rice, *Brachypodium*, *Ae. tauschii *and barley**. 1-5 indicate chromosomal segments, A-D, the putative breakpoints. The breakpoint B would be located within the ctg5 of *Ae. tauschii *physical map. The names on top are rice genes as listed in MSU Rice Genome Annotation (Osa1) Release 6.0.

The disruption of co-linearity between genomes is due to chromosomal rearrangements which occurred after the divergence of the two species from their common ancestor [17]. With the hypothesis of minimum chromosomal rearrangements occurring before speciation, we propose a model explaining the discontinuities in rice, *B. distachyon*, barley and *Ae. tauschii *synteny along the distal region of chromosome 3DS (Figure 3). The segment 5 (corresponding to ctg 17-24) was conserved between *Ae. tauschii *and barley while it appeared inverted between *Ae. tauschii *and rice/*B. distachyon*, and between barley and rice/*B. distachyon *(Figure 2). This proximal inversion probably took place in the *Triticeae *ancestor after its divergence from a common ancestor of *Oryzae *and *Brachypodium *lineages.

Additional rearrangements seemed to have occurred in the distal region after the divergence of the *Triticum/Aegilops *lineage from the *Hordeum *lineage. The ctg3-4-5 of the map showed inversions between *Ae. tauschii *and rice (LOC_Os01g02870-LOC_Os01g03160) (See Additional File 2). It partially overlapped the inversion observed between the barley region 8.9 cM-16.3 cM and the rice sequence LOC_Os01g03070-LOC_Os01g04020 (Figure 2). Thus, the segment 3 (ctg6-7) was collinear between rice/*Brachypodium *and *Ae. tauschii *while it was inside the inverted block between rice and barley. A translocation of the ctg3-distal ctg4 segment in a more proximal region in barley wouldn't explain the difference of synteny of the segment 3 between *Ae. tauschii*, barley and rice. A possible explanation would be some overlapping inversions with different chromosomal breakpoints in the *Triticum/Aegilops *and the *Hordeum *radiations (Figure 3). The block formed by segments 1 and 2 (corresponding to ctg3-4-5) would be inverted in the *Triticum/Aegilops *radiation, with the breakpoints A and C, while in *Hordeum*, the breakpoints may have occurred in B (within ctg4) and D leading to the inversion of a block formed by segments 2 and 3 (corresponding to a part of ctg4 and ctg5-6-7-8).

## Discussion

Beside the *Ph2 *locus, which is a suppressor of homoeologous chromosome pairing [13], other genes of interest have been previously mapped on chromosome 3DS including a resistance to tan spot [18], the brittle rachis gene [19], the *Gigantea *gene [20] and the developmental gene *Sphaerococcoid S1 *[21]. Some QTLs associated to yield components [22-24], spike length [25], black point resistance [26], resistance to *Fusarium *head blight *Fhb *[27], and grain protein content [28] have also been identified on 3DS. In addition, genes coding for several metabolic enzymes are located on 3DS: esterase 1, hexokinase-1,2, malic enzyme, peroxydase and triosphosphate isomerase [29]. Although a total of 57 molecular markers were found on chromosome 3D of the consensus genetic map of hexaploid wheat [30], the linkage group 3D usually shows only 10 to 20 markers for a specific segregating population [19,22,23,25]. This low density of markers makes the isolation of specific genes on the 770 Mb of chromosome 3D almost impossible. The difficulty could be overcome by using physical and genetic maps of *Ae. tauschii *[31].

We have used the *Ae. tauschii *genomic resources and a specific deletion bin (*ph2a*) to make the first physical map of part of a large plant genome based on global fingerprinting of the whole genome. The gaps in the map were evaluated by comparing the average CE along the region with the CE previously reported in wheat. If the average recombination is similar between wheat chromosomes, the difference observed would be due to a difference in physical distance. The average CE of the map was 2.35 cM/Mb, which is much higher than the maximum CE observed for all wheat chromosomes (0.87 cM/Mb) and for wheat chromosome 3BS (0.85 cM/Mb) [32,33]. The rate is about triple of previous reports, meaning that the actual physical distance would be 73 Mb, which is close to the estimated size of the *ph2a *deletion (80 Mb).

Comparison of the size of rice and *Ae. tauschii *short arm chromosomes reveals a genomic inference between the two species. The *Ph2 *map spanned 4.3 Mb of the distal region of rice short arm chromosome 1, from LOC_Os01g02090 to LOC_Os01g09580 [13]. The rice Os1S and wheat 3DS arms are respectively 17.1 Mb and 321 Mb long [6,34]. The increase of genome size between rice and *Ae. tauschii *would be 17× for the distal half (4.3 Mb in rice, 73 Mb in *Ae. tauschii*) and 19× for the proximal half of chromosome (12.8 Mb in rice, 248 Mb in *Ae. tauschii*). When rice genome was compared to sorghum genome, the genome size increase was entirely accounted for by the increase in the proximal heterochromatic regions [35]; the distal euchromatic regions were of identical lengths in the two species. The increase in genome size in the *Triticeae *is nearly equivalent in the distal and proximal regions, suggesting that the growth of *Triticeae *genomes took place along the entire chromosomes, in contrast to small sorghum genome where the growth took place only in the proximal region.

In plants, the recombination rate is higher in gene-dense regions and towards the telomeres and lower in centromeric regions [36], although reversals of this pattern exist. In wheat and barley, meiotic recombination rate follows a gradient along the chromosome arms decreasing from the telomere to the centromere [32,37]. The recombination rates along the 3DS region in *Ae. tauschii *dropped from 2.19 cM/Mb in the distal region to 0.09 cM/Mb in the proximal region. The CE in the distal and middle regions (2.19 and 1.42 cM/Mb) were calculated to be much higher for single contigs than the rate in the distal 3BS8 and 3BS7 bins (respectively 0.85 and 0.37 cM/Mb) [33]. In the proximal region, although the CE is overestimated due to the gaps between the contigs, the value is lower (0.09 cM/Mb) than the one of the orthologous bin 3BS9 (0.17 cM/Mb) [33]. Considering that the ctgs 17-22 were anchored on 3DS6-0.55-1.00 deletion bin (See Additional File 1), it means that recombination is almost null in half of the chromosome arm. While the recombination followed a gradient from telomere to centromere in barley, the *Ae. tauschii *genetic map showed alternated low and high- recombination regions along the short arm chromosome 3S, showing that the pattern of recombination might be chromosome specific.

Comparative studies between wheat, barley and model genomes such as rice and *Brachypodium *are helpful in understanding the structure of grass genomes and developing strategies to overcome linkage drag, which limits the introgression of wild genes in elite varieties [17]. As shown in previous studies, wheat chromosome 3 is largely collinear to rice Os1 [29,38] and to *B. distachyon *Bd2 [39]. The mid-scale comparative maps presented here revealed chromosomal rearrangements between *Brachypodium *and wheat that haven't been revealed in whole genome study [39]. Although the rice and *Brachypodium *genomes diverged 40-53 million years ago (Mya) and were largely rearranged from their common ancestor [39], the rice- and *Brachypodium*-*Ae. tauschii *co-linearity were similar with disruption in the same distal and proximal regions of *Ph2 *and 2 major inversions (Figure 3). Both inversions differentiating the 3DS physical map and the Os1 sequence have been detected in the comparison of the *Ae. tauschii *genetic map and rice and sorghum genome sequences and their origins were assigned to the *Triticeae *lineage [11], in agreement with inferences reported here. Additional *Ae. tauschii*-rice/*Brachypodium *rearrangements probably occurred but could not be detected in our map probably because of the low density markers on some contigs. For example, two inversions between rice chromosome 1 and 3BS reported in [6] were not observed in the *Ae. tauschii *3DS map due to the lack of markers on ctgs1-2-3.

The diploid genome of barley is usually considered as a good model for *Triticeae *genomics. Comparative studies using deletion bin, cytomolecular and low-resolution linkage maps showed a high level of synteny between wheat and barley chromosomes and revealed very few genomic rearrangements at the genome scale [40-43]. For example, the comparison of barley and *T. monococcum *revealed inversions and translocation between chromosomes 1H and 1A, and between chromosomes 5A and 4H [40]. As expected, the *Ae. tauschii *chromosome 3DS is largely collinear to the barley chromosome 3HS. However we reported here a disruption of the synteny for a distal region of 8.1 cM in barley, which has not been observed in previous studies.

The phylogeny of grasses suggests that the divergence of rice and *Brachypodium *from *Triticeae *occurred about 41-47 Mya and 32-39 Mya, respectively [39,44-46]. The structure of the orthologous *Ph2 *locus was almost identical in *Brachypodium *and rice. Although it implies that rice and *Brachypodium *are closer to each other than to wheat, DNA sequence would indicate rice and *Brachypodium*/*Triticeae *lineage split much earlier, with *Brachypodium *and the *Triticeae *splitting later as shown in [39]. The large proximal inversion of the segment 5 would have occurred after rice and *Brachypodium *diverged from a common ancestor of the *Triticeae*, leading to a collinear block between barley and *Ae. tauschii *(Figure 3).

The complexity of the rearrangements inside segment 5 between rice/*Brachypodium *and *Ae. tauschii *showed that the large inversion might actually be a series of inversions nested in each other. It might correspond to larger rearrangements between the adjacent bins 3S-0.57-0.78 and 3S-0.45-0.55 and rice chromosome 1 observed in the EST group 3 chromosome bin map [29]. Other inversion events have occurred in the distal region of *Ph2 *leading to different rearrangements of the rice/*Brachypodium*-barley-*Ae. tauschii *synteny. The disruption of co-linearity between wheat and barley showed that inversions would have occurred after the separation between *Hordeum *and *Triticum/Aegilops *species about 6-16 Mya [45]. However the inversions of segments 1 and 3 between barley and *Ae. tauschii *would need to be validated by increasing the resolution of the barley genetic map where the markers anchored on ctg 4 and 5, and ctg 6 and 7 co-segregated respectively at 8.2 cM and 12.5 cM.

## Conclusions

The construction of physical maps for a 5 Gb size genome is a complex and laborious task. Although the map reported here is only for a portion of the wheat genome, it is nevertheless the first report using fingerprinting of a complete *Triticeae *genome. It provided significant data showing chromosomal rearrangements between wheat, *Brachypodium *and barley, and it will be a useful resource for gene cloning of chromosome 3D. Whether or not the composition of *Triticeae *genomes with 90% of repeat DNA sequence [47] precludes the assembly of BAC contigs from whole-genome BAC libraries, this study showed that global fingerprinting of the large *Triticeae *genomes is capable of generating such physical maps despite the complexity of and high repeated sequence content in these genomes. This strategy is now used by the International Barley Sequencing Consortium which will soon release a physical map of barley [48].

## Methods

### Probe design and BAC filter hybridization

Plant material (*Triticum aestivum *cv Chinese Spring and nullisomic-tetrasomic derivatives) and the DNA extraction were respectively described in [13] and [49]. The BAC libraries and assembly of *Ae. tauschii *AL8/78 are described in [10] and http://wheatdb.ucdavis.edu.

The primers were designed to amplify unique 150-400 bp fragments of unigenes defined in the DFCI wheat gene index v11 http://compbio.dfci.harvard.edu/tgi by using Primer 3 program (See Additional File 3). The PCR reaction was carried out in 20 μl final volume with 400 nM of both primers, 200 μM of each dNTP, 1.5 mM of MgCl_2_, 0.5 U Taq Platinum polymerase (Invitrogen) with its 1× buffer and 40 ng of DNA. The PCR amplification followed a touch-down procedure: 1 cycle 2 min at 94°C, 10 cycles (20 sec 95°C, 20 sec Tm minus 0.5°C each cycle, 2 min 72°C), 35 cycles (20 sec 95°C, 20 sec Tm minus 5°C, 2 min 72°C) and an additional extension step of 10 min at 72°C (See Additional File 3). Probes were purified by cleaning up the PCR product or by electrophoresis on agarose gel using the MinElute kit (Qiagen) and verified by Sanger's method sequencing prior hybridization. We used the ABI PRISM^® ^BigDye™ Terminators V 3.1 for the sequencing reaction according to the manufacturer's instructions. The southern blot analyses onto membranes of restricted DNA from nullisomic-tetrasomic lines and onto *Ae. tauschii *BAC filters were performed according to [49] by pooling up to 8 probes. The positive BAC clones were cultivated in 96-well plates overnight in 200 μl of LB medium supplemented with 15 mg/l tetracycline for the BB and HB clones and with 15 mg/l chloramphenicol for HD, HI and RI clones. The cultures were centrifuged for 10 min at 5,000 rpm, and then the supernatant was replaced by MilliQ water. The anchoring of the markers on the positive BAC clones was validated by direct PCR with 1 μl of bacterial solution.

### Re- assembly of the BAC from targeted region

To minimize false assembly caused by genome-wide network, a subset of BAC clones including all clones of original contigs which contain positive clone(s) and singletons were re-assembled into contigs by using the FPC program [50,51]. The contig number(s) used were results of the re-assembly.

### Genetic mapping and recombination rate

For map alignments, wheat unigenes and markers sequences were compared to the 8× *B. distachyon *genome sequence http://www.brachypodium.org and to the rice pseudomolecules of Rice Genome Annotation Project - MSU Rice Genome Annotation (Osa1) Release 6.0 using BLASTN and TBLASTX. The homologues were assigned based on the best match to the rice pseudomolecules with significant threshold for the expectation E value of e-10. The same wheat sequences were used to identify the barley homologous unigenes of HarvEST:Barley (v1.76) assembly #35 by using BLASTN. The best matches were selected with a cut-off E value of e-20. The *Ae. tauschii *genetic map derived from the F2 population AL8/78 × AS75 [11] with additional markers described in Table S3. The genetic map of barley derived from the Steptoe x Morex doubled haploid population [52] was described in [15] and [16]. Recombination rate was calculated as a coefficient of exchange (CE) in cM per Mb.

## Authors' contributions

DF participated to the project design, designed the primers, assembled the BAC contigs based on EST anchoring, did the sequence alignment and the comparative maps, and drafted the manuscript. MCL designed the *Ae. tauschii *physical mapping research project and assembled the contigs based on fingerprinting. JD designed the *Ae. tauschii *physical mapping research project, analyzed the comparative maps and helped to draft the manuscript. LR analyzed the comparative maps and participated to the sequence alignment. BSG and ODA designed the *Ae. tauschii *physical mapping research project. FMY performed the fingerprint editing of the BAC libraries of *Ae. tauschii *and help to assemble contigs. ZS designed a subset of primers and performed the wet-lab work of BAC screening. KRD did the *Ae. tauschii *genetic mapping. PL designed and coordinated the research, analyzed the comparative maps and help to draft the manuscript. All authors read and approved the final manuscript.

## Supplementary Material

Additional file 1 **Description of the markers mapped onto *Ae. tauschii *physical map of distal region of chromosome 3DS**. Click here for file

Additional file 2 **Primer sequences and Tm used for the touch-down PCR of the wheat markers**. Click here for file

Additional file 3 **Physical map of the *Ph2 *locus in *Ae. tauschii *compared to the orthologous genomic sequence of rice Os1 and *B. distachyon *Bd2 (8× release)**. The names to the left are rice genes as listed in MSU Rice Genome Annotation (Osa1) Release 6.0. Click here for file
