# Supplementary material for: Physical mapping of a large plant genome using global high-information-content-fingerprinting: the distal region of the wheat ancestor Aegilops tauschii chromosome 3DS
Source: BMC Genomics. 2010 Jun 17;11:382. doi: 10.1186/1471-2164-11-382 (PMC2900270; doi:10.1186/1471-2164-11-382)
Supplement: Additional file 1 — Description of the markers mapped onto Ae. tauschii physical map of distal region of chromosome 3DS. [file 1471-2164-11-382-S1.DOCX]

| **Table S1**: Description of the markers mapped onto Ae. tauschii physical map of distal region of chromosome 3DS. | | | | | | | |  |  |  |  |  |
| --- | --- | --- | --- | --- | --- | --- | --- | --- | --- | --- | --- | --- |
| **BAC contig** | **marker** | **wheat unigenes (DFCI 11.0)** | **Locus** | **A bins** | **B bins** | **D bins** | **rice locus (TIGR 5.0)** | **rice protein function** | ***Brachypodium distachyon* (8x release)** | ***Ae. tauschii* genetic map (cM)** | **barley genetic map (cM)** | **barley molecular marker*** |
| 1 | 133 | TC286774 |  | 3AS4-0.45-1.00 | 3BS8-0.78-1.00 | 3DS3-0.24-0.55 | LOC_Os03g45320 | 3-isopropylmalate dehydrogenase 2, chloroplast precursor, putative | Bd5-27763077 |  |  |  |
| 2 | 151 |  |  |  |  |  | LOC_Os01g02800 | receptor-like kinase ARK1AS, putative | Bd2-814861 |  |  |  |
| 3 | 25 | TC321171 | *ph2a* |  |  |  | LOC_Os01g03160 | xylosyltransferase 1, putative | Bd2-1104649 |  |  |  |
|  | 146 | TC281750 |  |  |  |  | LOC_Os01g03144 | ABC-2 type transporter family protein | Bd2-1096844 | 17.08 | 15.55 | U35_6080 |
|  | 235 |  |  |  |  |  | LOC_Os01g03110 | expressed protein | Bd2-1168146 |  | 15.55 | 3_0113 |
|  | 173 |  |  |  |  |  | LOC_Os01g03110 | expressed protein | Bd2-911643 |  |  |  |
| 4 | 22 | TC294332 | *ph2a* |  |  |  | LOC_Os01g03070 | eukaryotic translation initiation factor 3 subunit 10, putative | Bd2-1062615 | 17.08 | 16.33 | 2_0172 |
|  | 24 | TC366721 |  |  | 3BS8-0.78-1.00 | 3DS6-0.55-1.00 | LOC_Os01g03100 | zinc finger family protein | Bd2-1074090 | 17.08 |  |  |
|  | 230 |  |  |  |  |  | LOC_Os01g03090 | putative S locus-linked protein SLL2 | Bd2-4146930 |  |  |  |
|  | 21 | TC296907 | *ph2a* |  |  |  | LOC_Os01g03060 | sarcoplasmic reticulum histidine-rich calcium-binding protein | Bd2-1055125 |  |  |  |
|  | 220 |  |  |  |  |  | LOC_Os04g02150 | Eukaryotic initiation factor 3, gamma subunit family protein |  |  |  |  |
|  | 222 |  |  |  |  |  | LOC_Os05g46520 | Polygalacturonase family protein | Bd2-1036918 |  |  |  |
|  | 20 | BE427255 | *ph2a* |  |  |  | LOC_Os01g03050 | fruit protein PKIWI502, putative | Bd2-1051720 |  |  |  |
|  | 140 |  |  |  |  |  | LOC_Os01g03040 | glycosyltransferase, putative |  |  |  |  |
|  | 135 |  |  |  |  |  | LOC_Os01g03040 | glycosyltransferase, putative | Bd2-1050139 |  |  |  |
|  | 125 | [TC305459](http://compbio.dfci.harvard.edu/tgi/cgi-bin/tgi/tc_report.pl?gudb=Wheat&tc=TC305459) |  |  |  | 3DS6-0.55-1.00 | LOC_Os01g02940 | glycosyltransferase, putative | Bd2-1036918 | 17.87 |  |  |
|  | 178 |  |  |  |  |  | LOC_Os01g02940 | glycosyltransferase, putative | Bd2-1022789 |  |  |  |
|  | 181 | TC331060 |  | 3AS4-0.45-1.00 | 3BS8-0.78-1.00 | 3DS6-0.55-1.00 | LOC_Os01g02930 | glycosyltransferase, putative | Bd2-911643 |  | 8.23 | 3_0297 |
|  | 182 |  |  |  |  |  | LOC_Os03g13220 | expressed protein | Bd1-67342097 |  |  |  |
|  | 183 |  |  |  |  |  | LOC_Os11g40590 | glycosyltransferase, putative | Bd4-13560079 |  |  |  |
|  | 134 |  |  |  |  |  | LOC_Os01g02920 | glycosyltransferase, putative | Bd2-881518 |  | 8.23 | 2_1190 |
|  | 238 |  |  |  |  |  | LOC_Os01g02910 | glycosyltransferase, putative | Bd2-18708955 |  |  |  |
|  | 176 |  |  |  |  |  | LOC_Os08g36320 | Glutamate decarboxylase, putative | Bd3-40374656 |  | 8.23 | 2_1398 |
| 5 | 245 |  |  |  |  |  | LOC_Os02g09540 | expressed protein | Bd3-4702984 |  |  |  |
|  | 116 | TC329855 | *ph2a* |  |  |  | LOC_Os01g02884 | ATP-dependent RNA helicase DHX36, putative | Bd2-852023 |  | 8.23 | U35_18435 |
|  | 131 |  |  |  |  |  | LOC_Os01g03320 | BBTI2 - Bowman-Birk type bran trypsin inhibitor precursor | Bd2-1305931 |  |  |  |
|  | 241 |  |  |  |  |  | LOC_Os10g32080 | Exostosin family protein | Bd3-29343700 |  |  |  |
|  | 240 |  |  |  |  |  | LOC_Os01g02880 | Fructose-bisphosphate aldolase 1, chloroplast | Bd2-841985 |  | 8.23 | 2_0529 |
|  | 18 | TC289464 | *ph2a* |  |  |  | LOC_Os01g02870 | nodulin MtN21 family protein, putative, expressed | Bd2-833144 | 22.78 |  |  |
| 6 | 124 | TC317798 |  | 3AS4-0.45-1.00 |  |  | LOC_Os01g03510 | 66 kDa stress protein, putative | Bd2-1269594 |  | 12.46 | 3_0915 |
|  | 27 | TC313624 |  | 3AS4-0.45-1.00 |  | 3DS6-0.55-1.00 | LOC_Os01g03520 | Ubiquitin-conjugating enzyme family protein | Bd2-1275517 | 25.42 | 12.46 | 2_0595 |
|  | 28 | TC310654 |  | 3AS4-0.45-1.00 |  | 3DS6-0.55-1.00 | LOC_Os01g03650 | sufB/sufD domain containing protein, expressed | Bd2-1291217 |  |  |  |
| 7 | WM1.1 | X81369 | *ph2a* |  |  |  | LOC_Os07g02450 | Leucine Rich Repeat family protein | Bd3-16774628 |  |  |  |
|  | WM1.2 |  | *ph2a* |  |  |  | LOC_Os07g02450 | Leucine Rich Repeat family protein |  |  |  |  |
|  | WM1.10 |  | *ph2a* |  |  |  | LOC_Os07g02450 | Leucine Rich Repeat family protein |  |  |  |  |
|  | WM1.11 |  | *ph2a* |  |  |  | LOC_Os07g02450 | Leucine Rich Repeat family protein |  |  |  |  |
|  | 29 | TC320738 | *ph2a* |  |  |  | LOC_Os01g03690 | serine/threonine-protein kinase receptor precursor, putative | Bd2-1312608 |  |  |  |
|  | 132 |  |  |  |  |  | LOC_Os01g03340 | Bowman-Birk type trypsin inhibitor mRNA | Bd2-1309046 |  | 12.46 | U35_7993 |
|  | 32 | TC344576 | *ph2a* | 3AS4-0.45-1.00 |  |  | LOC_Os01g03760 | expressed protein | Bd2-1345523 |  |  |  |
|  | isbp766 |  |  |  |  |  |  | - |  | 28 |  |  |
| 8 | BG262864 |  |  |  | 2BS | 2DS | [LOC_Os07g48310](http://blast.jcvi.org/er-blast/getSeq.cgi?id=LOC_Os07g48310%7C12007.t04464%7Cunspliced-genomic&db=/opt/www/blast/db/OSA1/Genes_in_TIGR_Rice_Pseudomolecules) | inactive receptor kinase At2g26730 precursor, putative | Bd1-14183362 |  |  |  |
|  | 34 | TC303152 |  |  | 3BS8-0.78-1.00 | 3DS6-0.55-1.00 | LOC_Os01g04190 | hexose transporter, putative, | Bd2-1535325 | 35.7 | 19.15 | 2_0742 |
|  | 35 | BE403480 |  | 3AS4-0.45-1.00 | 3BS8-0.78-1.00 | 3DS3-0.24-0.55 | LOC_Os01g04220 | DNA binding protein, putative | Bd2-1546219 |  |  |  |
|  | 36 | BE445620 | *ph2a* |  |  |  | LOC_Os01g04280 | calmodulin-binding protein, putative | Bd2-1576173 |  |  |  |
| 9 | 38 | BE499361 | *ph2a* |  |  |  | LOC_Os01g04630 | muconate cycloisomerase, putative | Bd2-1707529 |  | 21.68 | U35_6700 |
|  | BE495431 | [TC328226](http://compbio.dfci.harvard.edu/tgi/cgi-bin/tgi/tc_report.pl?tc=TC328226&species=wheat) |  |  |  | 3DS6-0.55-1.00 | LOC_Os01g46410 | leaf senescence related protein, putative | Bd3-22323948 |  |  |  |
|  | 39 | TC283704 | *ph2a* |  |  |  | LOC_Os01g04814 | AAA-ATPase-like protein mRNA | Bd2-1936860 | 42.4 | 24.22 | 2_0552 |
| 10 | 71 | TC281677 |  | 3AS4-0.45-1.00 |  | 3DS6-0.55-1.00 | LOC_Os01g08450 | GTP-binding protein YPTM2, putative | Bd1-22254424 | 46.9 | 24.99 | 2_0556 |
|  | 115 | TC286104 | *ph2a* |  |  |  | LOC_Os01g08450 | GTP-binding protein YPTM2, putative | Bd1-22244449 |  |  |  |
|  | 130 |  |  |  |  |  | LOC_Os01g02090 | expressed protein | Bd2-620637 |  |  |  |
|  | 174 |  |  |  |  |  | LOC_Os02g35440 | RING-H2 finger protein ATL4O precursor, putative | Bd3-47960632 |  |  |  |
| 11 | 42 | TC315365 | *ph2a* | 3AS4-0.45-1.00 | 3BS9-0.57-0.78 | 3DS6-0.55-1.00 | LOC_Os01g05010 | Mitochondrial glycoprotein | Bd2-2110092 | 48.17 | 28.44 | 1_1237 |
| 12 | 122 | TC339570 |  |  | 3BS9-0.57-0.78 | 3DS6-0.55-1.00 | LOC_Os01g05490 | Triosephosphate isomerase, cytosolic, putative | Bd2-2278555 | 53.91 | 32.83 | 1_0026 |
|  | 45 | TC319278 |  | 3AS4-0.45-1.00 | 3BS9-0.57-0.78 | 3DS6-0.55-1.00 | LOC_Os01g05500 | zinc finger in N-recognin family protein | Bd2-2293895 | 53.98 | 32.83 | 3_0571 |
| 13 | 161 | TC299086 | *ph2a* |  | 3BS9-0.57-0.78 |  | LOC_Os01g05840 | oxidoreductase, short chain dehydrogenase/reductase | Bd2-2493745 | 53.9 | 35.22 | 2_0492 |
|  | 58 | TC300397 | *ph2a* |  |  |  | LOC_Os01g07260 | expressed protein | Bd2-2890295 |  |  |  |
| 14 | 57 | BE500000 |  | 3AS4-0.45-1.00 | 3BS9-0.57-0.78 | 3DS6-0.55-1.00 | LOC_Os01g07250 | Saposin-like type B, region 1 family protein | Bd2-2883670 |  | 43.23 | 2_0666 |
|  | 58 | TC300397 | *ph2a* |  |  |  | LOC_Os01g07260 | expressed protein | Bd2-2890295 |  |  |  |
| 15 | 162 | TC347727 |  | 3AS4-0.45-1.00 | 3BS9-0.57-0.78 |  | LOC_Os01g07520 | proline-rich family protein, putative | Bd2-3036906 |  |  |  |
| 16 | 72 | TC279230 | *ph2a* |  |  |  | LOC_Os01g08540 | MutS domain III family protein | Bd2-3721848 |  |  |  |
| 17 | 85 | BE403509 |  |  | 3BS9-0.57-0.78 | 3DS6-0.55-1.00 | LOC_Os01g09550 | No apical meristem protein | Bd2-4180243 |  |  |  |
|  | 86 | TC332616 | *ph2a* | 3AS4-0.45-1.00 | 3BS9-0.57-0.78 | 3DS6-0.55-1.00 | LOC_Os01g09550 | No apical meristem protein | Bd2-4175918 |  |  |  |
|  | 87 | TC332477 |  | 3AS4-0.45-1.00 | 3BS9-0.57-0.78 | 3DS6-0.55-1.00 | LOC_Os01g09560 | Mitochondrial processing peptidase alpha subunit, putative | Bd2-4188507 | 66.18 | 47.09 | 2_0356 |
|  | 88 | TC292582 | *ph2a* |  |  |  | LOC_Os01g09580 |  |  | |  |  |
| 18 | 267 | [CJ896989](http://www.ncbi.nlm.nih.gov/entrez/viewer.fcgi?db=nucest&id=143607103) |  |  |  |  | LOC_Os08g34060 | DUF1336 domain containing protein | Bd3-39328878 |  |  |  |
|  | 62 | CV775439 |  |  |  | 3DS6-0.55-1.00 | LOC_Os01g07700 | expressed protein | Bd2-3128035 |  |  |  |
|  | 83 | TC292597 |  | 3AS4-0.45-1.00 | 3BS9-0.57-0.78 | 3DS6-0.55-1.00 | LOC_Os01g09300 | oxidoreductase, 2OG-Fe oxygenase family protein, putative | Bd2-4089256 | 66.55 |  |  |
|  | 84 | TC298539 | *ph2a* |  |  |  | LOC_Os01g09320 | NADP-dependent malic enzyme, chloroplast precursor, putative | Bd2-4097785 |  |  |  |
|  | 265 |  |  |  |  |  | LOC_Os01g09460 | Hexokinase 1, putative | Bd2-4146930 |  |  |  |
|  | [BJ259388](http://compbio.dfci.harvard.edu/tgi/cgi-bin/tgi/est_report.pl?GB=BJ259388&species=wheat) |  |  |  |  | 3DL | LOC_Os01g50750 | zinc finger, C3HC4 type domain containing protein | Bd2-47992195 |  |  |  |
|  | 567F1 |  |  |  |  | 5DL |  | - |  |  |  |  |
|  | FBA366 |  |  | 3AS |  |  |  | - |  |  |  |  |
| 19 | 74 | TC284450 |  |  | 3BS9-0.57-0.78 | 3DS6-0.55-1.00 | LOC_Os01g08814 | expressed protein | Bd2-3875051 | 66.6 | 48.63 | 2_1101 |
| 20 | 77 | TC302011 | *ph2a* |  |  |  | LOC_Os01g08970 | Structure-specific recognition protein 1 homolog | Bd2-3924449 |  |  |  |
|  | 79 | TC310618 | *ph2a* |  |  |  | LOC_Os01g09000 | Glutaminyl-tRNA synthetase, putative | Bd2-3953708 |  |  |  |
|  | 80 | TC300859 |  |  |  | 3DS6-0.55-1.00 | LOC_Os01g09010 | Transferase family protein | Bd2-3966290 |  | 48.63 | 2_0719 |
|  | 82 | BE517681 | *ph2a* |  | 3BS9-0.57-0.78 |  | LOC_Os01g09100 | WRKY DNA binding domain containing protein | Bd2-3983859 |  |  |  |
| 21 | 265 |  |  |  |  |  | LOC_Os01g09460 | Hexokinase 1, putative | Bd2-4146930 |  |  |  |
|  | 141 | TC299651 |  |  | 3BS9-0.57-0.78 | 3DS6-0.55-1.00 | LOC_Os01g02070 | pectinesterase inhibitor domain containing protein |  |  |  |  |
|  | 130 |  |  |  |  |  | LOC_Os01g02090 | expressed protein | Bd2-620637 |  |  |  |
| 22 | 119 | TC325987 |  | 3AS4-0.45-1.00 | 3BS1-0.33-0.57 | 3DS6-0.55-1.00 | LOC_Os01g08580 | Frigida, putative | Bd2-3755200 | 66.96 |  |  |
|  | FBB114 |  |  |  |  |  |  | - |  |  |  |  |
|  | PSR903 |  |  |  |  |  |  | - |  |  |  |  |
| 23 | 64 | TC315467 | *ph2a* |  |  |  | LOC_Os01g07740 | DEAD/DEAH box helicase family protein | Bd2-3140519 |  |  |  |
|  | 65 | TC281255 |  | 3AS4-0.45-1.00 | 3BS1-0.33-0.57 | 3DS6-0.55-1.00 | LOC_Os01g07760 | Phospholipase D alpha 1 | Bd2-3149686 |  | 49.41 | 1_1069 |
|  | 66 | BQ172292 | *ph2a* |  |  |  | LOC_Os01g07770 | peroxidase precursor, putative | Bd2-3155966 |  |  |  |
|  | BE404973 |  |  |  |  |  | LOC_Os11g47710 | SNF7 domain containing protein, putative | Bd4-8653804 |  |  |  |
|  | BE398525 |  |  |  |  |  |  | no |  |  |  |  |
| 24 | 68 | TC298000 |  | 3AS4-0.45-1.00 | 3BS1-0.33-0.57 | 3DS6-0.55-1.00 | LOC_Os01g08150 | expressed protein | Bd2-3400868 |  |  |  |
| * POPA name and HarvEST:Barley v1.68 vs the assembly #35 of the Steptoe x Morex genetic map with gene-based SNP (14, 15) | | | | | | | |  |  |  |  |  |
