# Supplementary material for: Physical mapping of a large plant genome using global high-information-content-fingerprinting: the distal region of the wheat ancestor Aegilops tauschii chromosome 3DS
Source: BMC Genomics. 2010 Jun 17;11:382. doi: 10.1186/1471-2164-11-382 (PMC2900270; doi:10.1186/1471-2164-11-382)
Supplement: Additional file 2 — Primer sequences and Tm used for the touch-down PCR of the wheat markers. [file 1471-2164-11-382-S2.DOCX]

| **Table S2**: Primers sequences and Tm used for the touch-down PCR of the wheat markers. | | | | |
| --- | --- | --- | --- | --- |
|  |  |  |  |  |
| **marker** | **wheat unigene** | **wheat F primer** | **wheat R primer** | **Tm** |
| 71* | TC281677 | CTATGATGTGACAGACCAAGATAGC | CCATGAAAGCCTGTTCAACATT | 62 |
| 133 | TC286774 | AGACGTTGGACAATGGGTTC | TATCTATGCGGGCTTTCGAC | 67 |
| 25 | TC321171 | GAAGAATCCAAGTCAAGCAACAG | ATTACACTCCCTACAAGCTCAGGTA | 63 |
| 24 | TC366721 | AATTCTGTTTCCGATCTTGGTGA | TGCATCAGAAAATAGTATACGCAGA | 59 |
| 22 | TC294332 | GTGTAGATCTAAGGAAGGGCAGATT | GTATGTCTCCCACAGAAACTTGAAC | 63 |
| 21 | TC296907 | AGAAGGCACAGGAAACATGG | ACCCTTCGCTCATCATCATC | 66 |
| 20 | BE427255 | GTCCAATCCGGTCACTCATT | CAGTCCACTGCTCATCTGGT | 62 |
| 125 | [TC305459](http://compbio.dfci.harvard.edu/tgi/cgi-bin/tgi/tc_report.pl?gudb=Wheat&tc=TC305459) | TTGCAAGATGGCGACTGAGAAGAAAAT | GCCTTGCATTCCGGAACGTG | 63 |
| 181 | TC331060 | CGTACGGGAACCTAGAGCAC | TCACTTGAGGTAAATAAATCAACCA | 60 |
| 116 | TC329855 | TTATCGCTCTTCGCCTGACT | AACCCGTCCTACAAGACGAA | 59 |
| 18 | TC289464 | TTCACCGAGGAGGACATGAG | GATGATCCCTCCGGTGTAGA | 59 |
| 124 | TC317798 | TCAGCAACCTGAATTTGCAC | ATAGGACCCCGGTGTTTTTC | 63 |
| 27 | TC313624 | TCAGAATCTGGCACTAGTGTATCAA | GATTCCATAGGATGTGAAACAGATG | 63 |
| 28 | TC310654 | TTGTGGTACAGGAAATGGTATTTTT | CAGGGAAACATGTATATGTAACCAAA | 57 |
| WM1.1 | NP234786 | CTGGTGCAATACCTCGGCAT | TATGATATATAAGTTGCTGC | 62 |
| 29 | TC320738 | ATTACACATGGAAACTGTCAGAGGA | AGTCCCAGACTAACAACACTGTCTC | 60 |
| 32 | TC344576 | GGGAGAATTTGTTTTGTGGTGTAT | TCCTGTCCAAGATATTTACTGTTCC | 65 |
| 34 | TC303152 | GGATGTTGTCTCTGCCTTTTATGTA | AATATAATTACACATCACGCTTTGAA | 60 |
| 34* | TC303152 | TACTCTACATCAGTGTTCCGCAGT | AGGACAATGACAAGAGTAACATCG |  |
| 35 | BE403480 | GCTGTCCGAGTCGTGCAT | TAGTAGCAGTCGCCGTAGCC | 62 |
| 36 | BE445620 | AAGGAGGGTGCCTTCCAC | GGCAGAGGGAGTTGACGTAG | 64 |
| 38 | BE499361 | GAGTGGAGATGGCAGTGATTG | ACTTCAATTGCTTGGTTTGCTGT | 63 |
| 39* | TC283704 | TCTGTACACTCTGGTAGCCCTTACT | TAGAACTATACAAGCTGCACCACAA | 68 |
| 42 | TC315365 | GTTGAGGAGATCCCAGATAGCTT | CAGGTGAATTCGAGACTTGGAC | 57 |
| 122 | TC339570 | TGAGATGCTTGTCAACCTCG | CTGTTCAGCGACAACTTCCA | 62 |
| 45 | TC319278 | TTTCAAATTCAGCCACTGGA | CAATCAGGGCATTGCATCTT | 61 |
| 161* | TC299086 | GATGTTCTGAAGCTTGACCTTAGC | TATCAAGGAGAAGGTTGGTCAGTAG | 66 |
| 58 | TC300397 | CACTTGACATTTGATGAAACTCTCTT | TGGTAAATCTCAGGTTCAGAAGACT | 63 |
| 57 | BE500000 | AGAGCAGAGATGCATATATGAGGAG | GTTTAACCACAGAACTCCAAGAAGA | 65 |
| 58 | TC300397 | CACTTGACATTTGATGAAACTCTCTT | TGGTAAATCTCAGGTTCAGAAGACT | 63 |
| 162 | TC347727 | AATATTCACAACAACCTCAGCCTAC | CAGAAAAGCTACTTCCTCCCTGT | 60 |
| 72 | TC279230 | GTATCGTTCATATCCAATTCCTTTG | CAATGAGCTGATTTCACCTTCTTAT | 63 |
| 85 | BE403509 | CCTAAGCAAGCATTTCTTCCATC | GTAGAAGATCTTGCAAACGACGAG | 63 |
| 86 | TC332616 | AGGAGGTTGGTATAGGTGCTGAT | AATTTGTCGTGGTGGAGAAAAAT | 61 |
| 87 | TC332477 | ATGCCTGAATGGTGGAAGTG | CAGACAGGAGTGGTTCAGCA | 63 |
| 88 | TC292582 | GTCATATTGTGGCTTTGAAAGTTCT | CAACTCCTTGTACAGCTCTCCTTT | 59 |
| 74* | TC284450 | CCTCGGACCAAACTTGTTGA | ATTCCACAACCAAAGCAAGG | 64 |
| 62 | CV775439 | GGCCCTGGCCTGCTACTT | AGTGTAGTAAAGGAAGACGCTCCAT | 63 |
| 83 | TC292597 | TGCTTGGATTCAGGCTTCTT | CCATTCACTTGGTTTTCTGGA | 63 |
| 84 | TC298539 | TGGAGATCAATTTTGCAGTAATAAA | GCTCTGTGTCCTTATTACACTCGTT | 59 |
| 77 | TC302011 | GATGTAAGCAGCCTGATCAATTTC | CGAGTCTTTCTCATTAGCACCAGTA | 61 |
| 79 | TC310618 | AATCAAGGAATACAGGGAAAAGAAG | AGGCAACAAGTAGCCAGTAGTATGA | 63 |
| 80 | TC300859 | CGCTCAAGACGCAGTACAAG | ATCATGTCGATAACCTCCATCAC | 62 |
| 82 | BE517681 | CGTAGCTCGTCTGTGCAGT | GAGTAGTCGGAGGCACGAG | 60 |
| 141 | TC299651 | AGCCACTAGTGCCCTTGAGA | TGCGGACAAGTAGGTTGATG | 62 |
| 119 | TC325987 | CAAGGCTGTCATCAGGTGTG | CGCATAGTAGGGCTGAGCA | 60 |
| 64 | TC315467 | ATCTACTTGTCTATATGGCGGTGCT | ACATGCTGGGTGATTGACTTATTT | 61 |
| 65 | TC281255 | CAAGAGAGCCATGGCATGAT | CAGCAGTAGGAACTTCCAAGG | 59 |
| 66 | BQ172292 | GGGTTGCCCACCAATCAAT | AGGGGGTGGTGCAGAAGTA | 61 |
| 68 | TC298000 | TGTTGTGTACAAGGAACAACTCA | TAACTCATTGTTGAAGTCCCTTCTC | 59 |
| * primers used for genetic mapping (gene-based SNP) | | |  |  |
