# Supplementary material for: Physical mapping of a large plant genome using global high-information-content-fingerprinting: the distal region of the wheat ancestor Aegilops tauschii chromosome 3DS
Source: BMC Genomics. 2010 Jun 17;11:382. doi: 10.1186/1471-2164-11-382 (PMC2900270; doi:10.1186/1471-2164-11-382)
Supplement: Additional file 3 — Physical map of the Ph2 locus in Ae. tauschii compared to the orthologous genomic sequence of rice Os1 and B. distachyon Bd2 (8× release). The names to the left are rice genes as listed in MSU Rice Genome Annotation (Osa1) Release 6.0. [file 1471-2164-11-382-S3.PPT]

## Slide 1
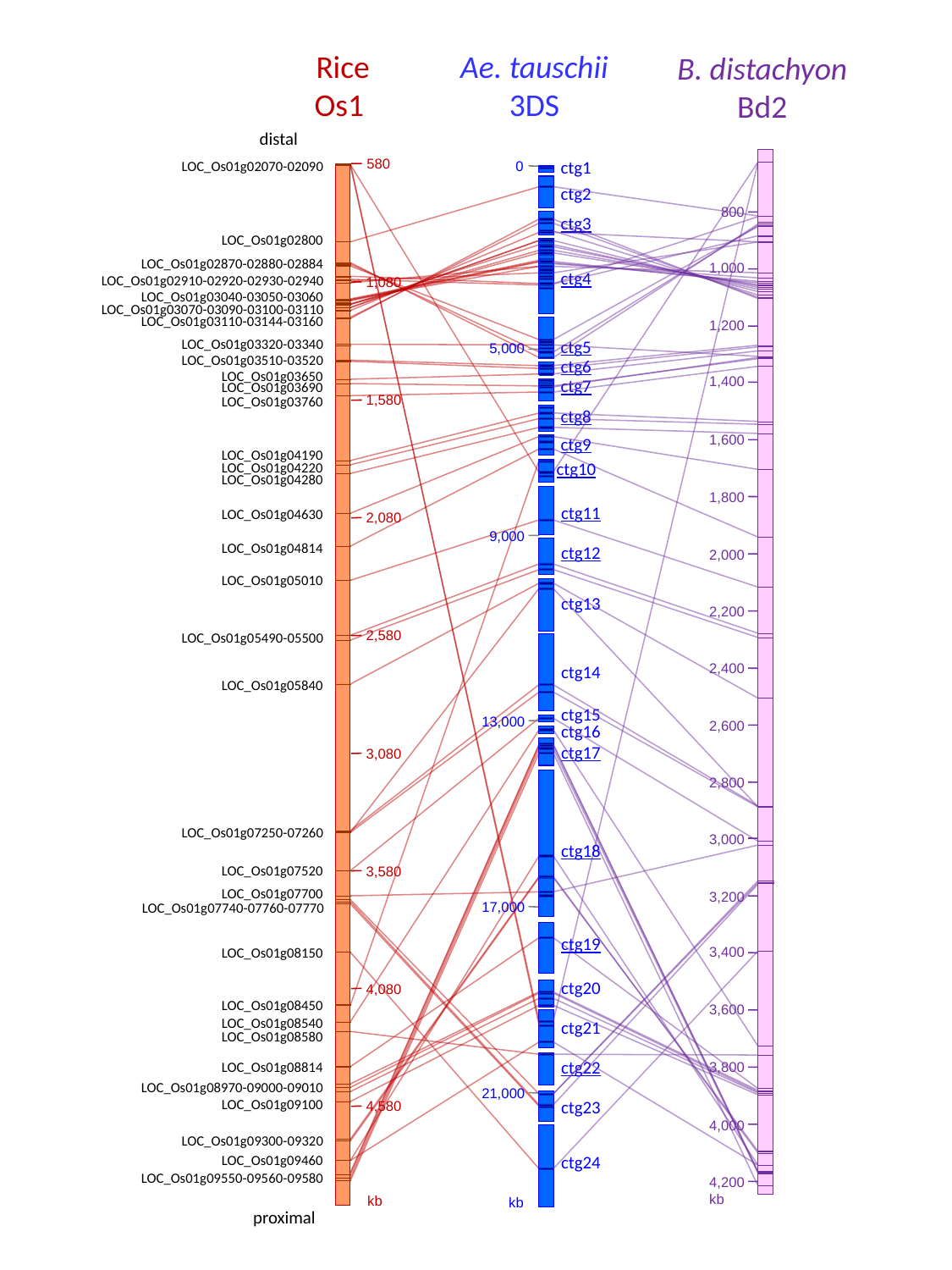

Rice
Os1
Ae. tauschii
3DS
B. distachyon
Bd2
distal
800
1,000
1,200
1,400
1,600
1,800
2,000
2,200
2,400
2,600
2,800
3,000
3,200
3,400
3,600
3,800
4,000
4,200
kb
ctg1
0
5,000
9,000
13,000
17,000
21,000
kb
LOC_Os01g02070-02090
580
1,080
1,580
2,080
2,580
3,080
3,580
4,080
4,580
kb
ctg2
ctg3
LOC_Os01g02800
LOC_Os01g02870-02880-02884
ctg4
LOC_Os01g02910-02920-02930-02940
LOC_Os01g03040-03050-03060
LOC_Os01g03070-03090-03100-03110
LOC_Os01g03110-03144-03160
ctg5
LOC_Os01g03320-03340
LOC_Os01g03510-03520
ctg6
LOC_Os01g03650
ctg7
LOC_Os01g03690
LOC_Os01g03760
ctg8
ctg9
LOC_Os01g04190
ctg10
LOC_Os01g04220
LOC_Os01g04280
ctg11
LOC_Os01g04630
LOC_Os01g04814
ctg12
LOC_Os01g05010
ctg13
LOC_Os01g05490-05500
ctg14
LOC_Os01g05840
ctg15
ctg16
ctg17
LOC_Os01g07250-07260
ctg18
LOC_Os01g07520
LOC_Os01g07700
LOC_Os01g07740-07760-07770
ctg19
LOC_Os01g08150
ctg20
LOC_Os01g08450
LOC_Os01g08540
ctg21
LOC_Os01g08580
ctg22
LOC_Os01g08814
LOC_Os01g08970-09000-09010
LOC_Os01g09100
ctg23
LOC_Os01g09300-09320
ctg24
LOC_Os01g09460
LOC_Os01g09550-09560-09580
proximal
